# The relationship between diverticulosis and colorectal neoplasia: A meta-analysis

**DOI:** 10.1371/journal.pone.0216380

**Published:** 2019-05-29

**Authors:** Hyun Jung Lee, Soo Jung Park, Jae Hee Cheon, Tae Il Kim, Won Ho Kim, Hyun Jung Kim

**Affiliations:** 1 Department of Internal Medicine and Liver Research Institute, Seoul National University College of Medicine, Seoul, Korea; 2 Department of Internal Medicine, Severance Hospital, Yonsei University College of Medicine, Seoul, Korea; 3 Department of Preventive Medicine, College of Medicine, Korea University, Seoul, Korea; University Hospital Llandough, UNITED KINGDOM

## Abstract

**Background/Aims:**

Diverticulosis and colorectal neoplasia share epidemiological trends and risk factors which are common in Western countries and incidences increase with age. However, the data on an association between diverticulosis and colorectal neoplasia are conflicting. Thus, we performed a meta-analysis to evaluate whether diverticulosis is associated with colorectal neoplasia.

**Methods:**

A systematic literature search of PubMed, EMBASE, Cochrane Library, Web of Science, and SCOPUS was conducted to identify studies that investigated the association between diverticulosis and advanced colorectal neoplasia (advanced adenoma, colorectal cancer), adenomas, or polyps. The demographic characteristics of patients, including age, gender, indication for colonoscopy, confounding factors, and outcomes of colorectal neoplasia were assessed.

**Results:**

We identified 29 cross-sectional studies (N = 450,953) that investigated the association between diverticulosis and colorectal neoplasia. The meta-analysis found that diverticulosis was not associated with advanced colorectal neoplasia (odds ratio [OR] 0.98, 95% confidence interval [CI] 0.63–1.50). Although there was a positive correlation between diverticulosis and adenomas (OR 1.47, 95% CI 1.18–1.84) and diverticulosis and polyps (OR 1.95, 95% CI 1.15–3.31), diverticulosis did not increase the risk of adenomas (OR 1.34, 95% CI 0.87–2.06) in patients who underwent screening colonoscopy. Moreover, all the increased risk of colorectal neoplasia in patients with diverticulosis was observed in published studies only, and not in unpublished ones.

**Conclusions:**

This meta-analysis demonstrated that diverticulosis is not associated with an increased risk of advanced colorectal neoplasia. Although diverticulosis was associated with a higher risk of polyps and adenomas, the risk was not increased in screening populations. Moreover, the increased risk of colorectal neoplasia in patients with diverticulosis was observed only in published studies and not in unpublished ones.

## Introduction

Colonic diverticulosis is the condition of outpouching of the colonic mucosa and submucosa as a result of weakness in the muscle layers of the colonic wall. Patients with diverticulosis are usually asymptomatic; only 15%~25% of patients with diverticulosis develop complications such as diverticulitis and gastrointestinal bleeding [1]. It is uncommon before the age of 40, with an estimated prevalence of 5%, which increases up to 65% of people aged 65 years and older [2, 3]. Similar to diverticulosis, the prevalence of colorectal cancer (CRC) also begins to increase in people aged between 40 and 50 years, and the age-specific incidence rates increase with each increasing decade [4]. Because the prevalence of diverticulosis and colorectal neoplasia markedly increases over the last decades [3], many studies have been conducted to identify risk factors and develop risk reduction strategies.

Diverticulosis and colorectal neoplasia share risk factors that are common to Western countries, such as smoking, alcohol, constipation, and low fiber diet [3, 5, 6]. The lack of dietary fiber and slow colonic transit times have been suggested to be involved in the pathogenesis of both conditions [6–8]. Although both diverticulosis and colorectal neoplasia show epidemiological trends and risk factors in common, the data on diverticulosis and colorectal neoplasia have not yet confirmed an association. While many studies have found a high prevalence of adenomas in patients with diverticulosis [9–13], others have failed to confirm that observation [14]. In addition, with regard to advanced colorectal neoplasia including CRC, some studies found a higher prevalence of advanced colorectal neoplasia in patients with diverticulosis compared with controls [10, 15], whereas others failed to show a significant difference [9, 16, 17]. Therefore, in this study, we performed a systematic review and meta-analysis of currently available studies to evaluate the association between diverticulosis and colorectal neoplasia.

## Materials and methods

We used multiple comprehensive databases to identify literature investigating the association between diverticulosis and advanced colorectal neoplasia (advanced adenoma, CRC), adenomas and polyps. This study is based on the Cochrane Review Methods [18].

### Data & literature sources

We searched MEDLINE (1964 to Jan 2019), EMBASE (1947 to Jan 2019), the Cochrane Central Register of Controlled Trials (1966 to Jan 2019), Web of Science (1964 to Jan 2019), and SCOPUS (1964 to Jan 2019). We did not restrict our search with regard to language or year of publication. The following keywords and medical subject headings (MeSH) were searched via MEDLINE: (“Diverticulosis, Colonic” or “Diverticulum” or “Diverticulum, Colon”) AND (“Colorectal Neoplasms” or “Cecal Neoplasms” or “Colonic Polyps” or “Adenoma, Villous” or “Adenomatous Polyps”). See S1 Appendix for the comprehensive list. The search strategies adapted for other databases were based on the MEDLINE strategy. After the initial electronic search, we performed searches by hand for additional relevant articles from the bibliographies of identified studies. Articles identified were assessed individually for inclusion.

### Study selection

The inclusion of all studies was independently decided by two reviewers (HJL and HJK) based on the selection criteria. Study selection was performed through 2 levels of screening: At the first level, we screened titles and abstracts of identified studies. At the second level, we screened the full text. Studies were included in our meta-analysis if they satisfied the following: (1) diverticulosis documented by colonoscopic examination; (2) the outcomes reported including advanced colorectal neoplasia (advanced adenoma, CRC), adenomas, and polyps; (3) relative risks (RR) or odds ratio (OR) reported or data provided for their calculation; and (4) were cohort, cross-sectional and case-control studies, but neither case reports nor reviews. Neither type of publication nor language were restricted. Articles were excluded from this meta-analysis if the published data were insufficient for estimating the relative risks/odds ratios (RRs/ORs) and 95% confidence intervals (CIs).

### Data extraction

The two reviewers independently extracted data from each study using a predefined data extraction form. Any disagreement unresolved by discussion was then reviewed by the third author (SJP). The following variables were extracted from studies: the first author; journal name; year of publication; study design; country/ethnicity of study participants; publication type; study period; total number of patients; age; gender; indication for colonoscopy (screening vs. diagnosis of symptoms); confounding factors; outcomes including the presence of advanced colorectal neoplasia (advanced adenoma, CRC), adenomas, and polyps; and RR/OR and 95% CIs. Diverticulosis was defined as the presence of endoscopically diagnosed diverticuli in any part of the colon. Advanced colorectal neoplasia consisted of advanced adenoma and/or CRC. An advanced adenoma was defined as a tubular adenoma ≥1cm in size, any polypoid lesion with a villous histology, high grade dysplasia. A polyp was defined as any localized projection above the surrounding colonic mucosa regardless of histologic evaluation. Colonic segments proximal to the splenic flexure, which included the cecum, ascending, and transverse colon, were defined as proximal colon; and portions of the colon distal to the splenic flexure, including the descending and sigmoid colon, and rectum, as distal colon. If any of the variables listed in this section were not mentioned in a study, we requested the data via email.

### Assessment of methodological quality

Two reviewers (HJL and HJK) independently assessed the methodological quality of each study, using the appraisal tool for cross-sectional studies (AXIS tool) [19]. Any unresolved disagreement between the reviewers was resolved by a discussion with or review from the third author (SJP). The AXIS tool consisted of 20 questions in total 5 parts: introduction, methods, results, discussion, and other.

### Statistical analysis

The primary outcome of our review was to determine the risk of advanced colorectal neoplasia (advanced adenoma and/or CRC) based on the presence of diverticulosis. As a secondary outcome, we also evaluated the associations between diverticulosis and adenomas and polyps. We conducted pooled analyses using the inverse variance method, with random-effects weighing for meta-analyses of outcomes reported by multiple studies that were sufficiently similar to justify combining results. To estimate heterogeneity, we estimated the proportion between-study inconsistency due to true differences between studies (rather than differences due to random error or chance) using *I*^2^ statistic, with values of 25%, 50%, and 75% considered low, moderate, and high, respectively. If the data were available, we used effect sizes from multivariate models, with confounding factors adjusted in each study. We performed subgroup analysis to assess the association between diverticulosis and adenomas and polyps according to the specific indication for colonoscopy (screening vs. diagnosis of symptoms).

We performed sensitivity analysis after excluding studies with low methodological quality or abstract-only studies to check whether the results had changed. If the results did not change significantly after excluding low-quality studies (abstract-only study), then they were considered to be robust. If the results had changed or the conclusions differed, then they had low stability.

The Egger test was used to evaluate publication bias, with *P* < 0.05 suggesting a significant publication bias. We used RevMan version 5.2 (Copenhagen, Denmark) and Stata 13.1/MP version for these analyses. An OR of >1 favored the risk factor and *P* < 0.05 and a 95% CI that did not include the value 1 were considered to be statistically significant.

## Results

### Identification of studies

**Fig 1:**
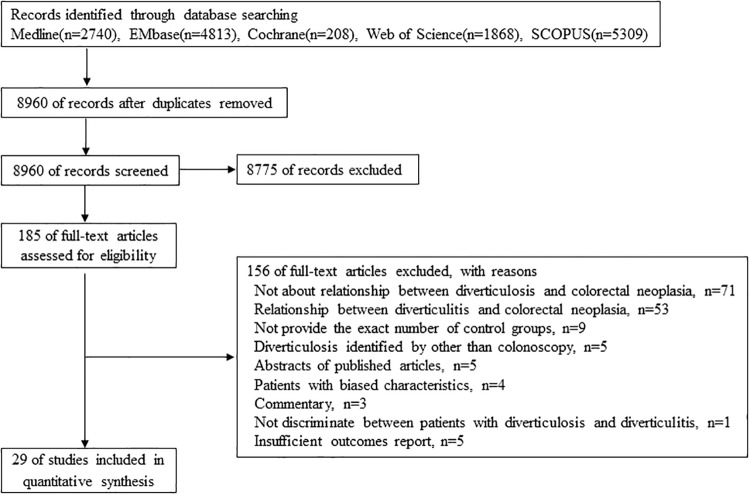
Flow diagram for determining study inclusion.

A flow diagram of our review is shown in Fig 1. Searches of the databases resulted in 8960 articles. Of these, 8775 publications were excluded based on the title and abstract which did not fulfill the selection criteria. We obtained the complete manuscripts of the remaining articles, and following scrutiny of these, we identified 185 potentially relevant studies. We excluded 156 articles for the following reasons: 71 were not about the relationship between diverticulosis and colorectal neoplasia, 53 were about the relationship between diverticulitis and colorectal neoplasia, 9 did not provide the exact number of control groups, 5 identified diverticulosis by methods other than colonoscopy, 5 were abstracts of published articles, 4 included patients with biased characteristics, 3 was commentary, 1 did not discriminate between patients with diverticulosis and diverticulitis, and 5 reported outcomes that were insufficient for our aims. The final total number of studies included in our meta-analysis was 29 [9–13, 15–17, 20–40].

### Study characteristics and patient populations

A total of 29 cross-sectional studies (N = 450,953) investigated the association between diverticulosis and colorectal neoplasia. We used a standardized protocol and report form to abstract the following data from each publication: first author, year of publication, country/ethnicity, publication type, study period, total number of patients, prevalence of diverticulosis (%), mean age, male (%), confounding factors, and reported outcomes. The studies were published between 2000 and 2019, with an enrollment period ranging from 1995 to 2018. Among the 450,953 participants analyzed in this meta-analysis, 32,235 (7.1%, range 0.6 ‒ 54.1%) had diverticulosis, 53.4% were male, and the mean age was 56.0 years. In addition, 19 studies were performed in Western countries and 9 studies in Asia. Of these 29 studies, 16 reported data on advanced colorectal neoplasia, 19 on adenomas, and 14 on polyps. The characteristics of these studies are summarized in Table 1.

**Table 1:** Demographic characteristics of included studies.

Study	Year	Country/ethnicity	Publication type	Study period	Total number (No of diverticulosis)	Diverticulosis (%)	Mean age (range)	Male (%)	Confounding factors	Outcome
Yusuf	2000	Pakistan	Abstract	1995–1999	311 (2)	0.6	45.3 (5–91)	186 (60)		CRC
Loffeld	2002	Netherland	Article	n/r	6827 (1849)	27.1	56.6	2901 (42.5)		Polyp, CRC
Morini	2002	Italy	Article	2000	630 (291)	46.2	62.9	326 (51.7)		Adenoma, Advanced adenoma, CRC
Rajendra	2005	Malaysia	Article	2000–2003	410 (41)	10	51.7 (12–91)	217 (52.9)	Age, ethnity, and symptoms	Adenoma, CRC
Choi	2007	South Korea	Article	2002–2004	2377 (215)	9	50.8	1355 (57)		Adenoma, Advanced neoplasia
Rangnekar	2007	USA	Abstract	n/r	589 (308)	52.3	60.6	n/r		Adenoma
Hirata	2008	Japan	Article	2006–2007	672 (165)	24.6	50	204 (59.8)	Age and gender	Polyp
Hammoud	2009	USA	Abstract	n/r	1668 (899)	53.9	n/r	n/r		Polyp, Adenoma, Advanced adenoma, CRC
Lee	2010	South Korea	Article	2008–2009	1030 (203)	19.7	52.5 (19–86)	611 (59.3)	Age, gender, alcohol, smoking, BMI, and co-morbidity	Polyp, Advanced neoplasia
Mazumder	2011	N/A	Abstract	2009	1000 (272)	27.2	57.3	400 (40)		Polyp
Neubauer	2011	Poland	Abstract	n/r	3011 (425)	14.1	52.5 (17–100)	1235 (41.0)		Adenoma, CRC
Rondagh	2011	Netherland	Article	2008–2009	2319 (855)	37	58.4	1065 (46.1)		Polyp, Adenoma, Advanced neoplasia
Szura	2011	Poland	Abstract	n/r	22441 (5360)	23.9	55.1 (16–95)	9331 (41.6)		Polyp, CRC
Gohil	2012	USA	Article	n/r	142 (40)	28.2	52 (40–70)	54 (38.0)	Gender and bowel preparation	Polyp, Adenoma
Azzam	2013	Saudi Arabia	Article	2007–2010	3649 (270)	7.4	60.8 (12–110)	2230 (61.1)		Adenoma
Parava	2013	USA	Abstract	2011–2012	1077 (512)	47.5	57	592 (55)		Polyp, Adenoma, Advanced adenoma
Meda	2014	USA	Abstract	n/r	890 (313)	35.2	n/r	n/r		Adenoma
Muhammad	2014	USA	Article	2009–2011	2223 (1203)	54.1	61.2	2074 (93.3)	Age and indication of colonoscopy	Polyp
Shen	2014	USA	Abstract	2009–2010	1363 (302)	22.2	59.3	665 (48.8)	Age, gender, ethnity, alcohol, smoking, BMI, and co-morbidity	Adenoma
Ashktorab	2015	USA	Article	2012	1986 (702)	35.3	57 (18–92)	867 (43.6)	Age and gender	Polyp, Adenoma
Peery	2015	USA	Article	2013–2015	624 (260)	41.7	54	271 (43.4)		Adenoma, Advanced adenoma
Wang	2015	Taiwan	Article	2009–2011	1899 (256)	13.5	52.8 (20–86)	1203 (63.2)	Age and alcohol	Adenoma
Wong	2016	Brunei	Article	2011–2014	2766 (479)	17.3	53.2	1434 (51.8)		Polyp, CRC
Shah	2017	USA	Abstract	n/r	896 (420)	46.9	n/r	n/r	Age and gender	Adenoma
Teixeria	2017	Portugal	Article	2013–2014	203 (62)	30.5	65.5	95 (47)		Adenoma
Hong	2018	China	Article	2013–2014	17456 (424)	2.4	49.1	10146 (58.1)	Age and gender	Adenoma
Pavao	2018	Portugal	Abstract	2011–2016	414 (207)	50	63.5	206 (49.8)		Adenoma, Advanced adenoma
Rodriguez	2018	Italy	Abstract	2009–2018	25962 (7936)	30.6	60.9 (18–102)	12959 (50)		Polyp, CRC
Wang	2019	China	Article	2000–2016	346118 (7964)	2.3	56 (11–92)	188067 (54.3)		Polyp, CRC

BMI, body mass index; A, adenoma; P, polyp, AA, advanced adenoma, CRC, colorectal cancer, AN, advanced neoplasia, n/r, not clear

### Quality of the included studies

The quality assessments of the individual studies are listed in S1 Table which were not available in abstract only studies. All the included studies were cross-sectional studies, so they could only show an association but not demonstrate causality. Overall, the studies seemed to address study design and reporting quality as well as risk of bias appropriately. However, though most studies clearly defined the reference population and the sampling frame representative of the target population, 11 studies (64.7%) did not present the exact number of patients who were initially enrolled and excluded through the selection process. In addition, 9 studies (53.0%) were not adequately accounted for important potential confounding factors such as age, gender, and comorbidities, which could affect the prevalence of colorectal neoplasia.

### Diverticulosis and advanced colorectal neoplasia

**Fig 2:**
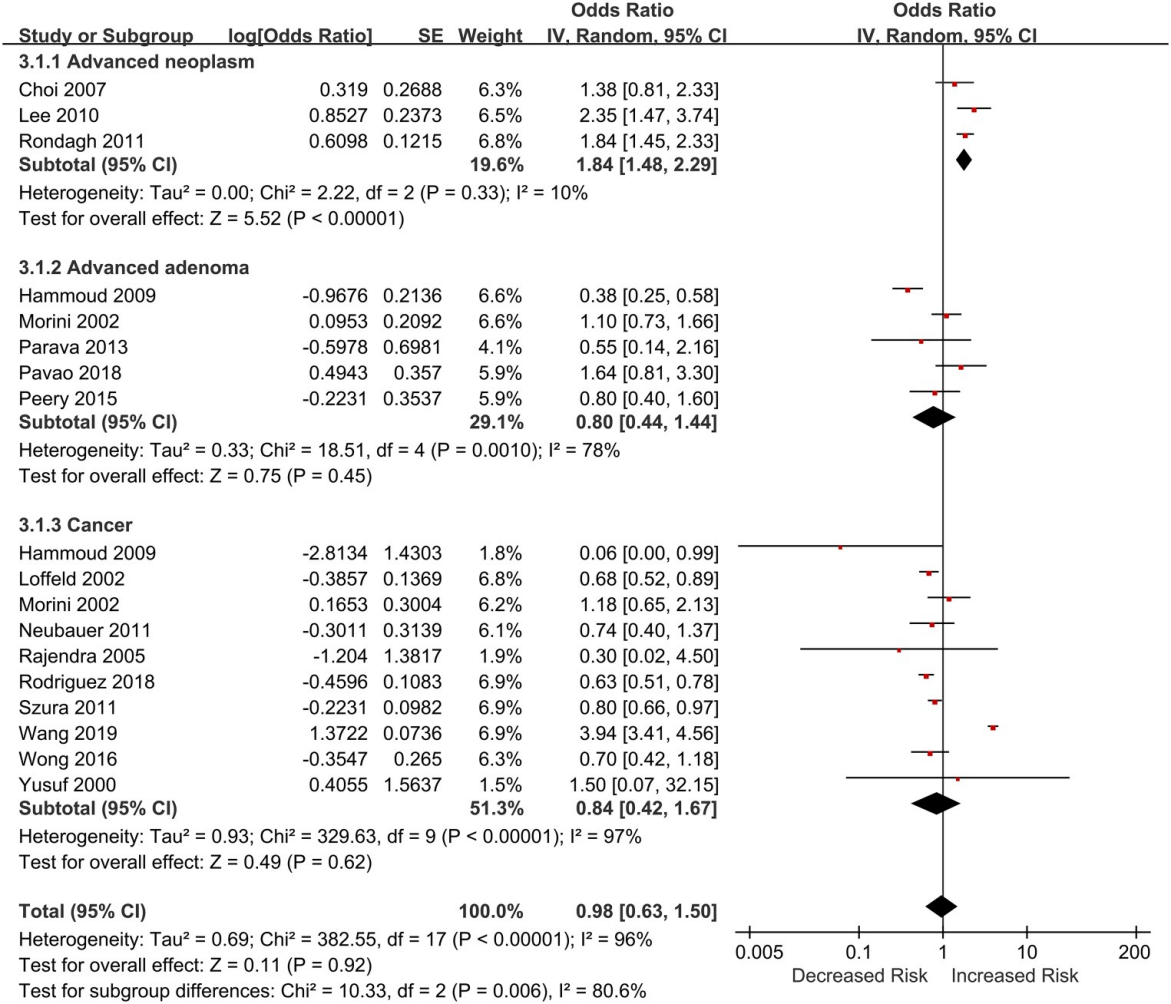
Forest plots of diverticulosis with advanced colorectal neoplasia.

Sixteen studies investigated the association between diverticulosis and advanced colorectal neoplasia, including 3 advanced neoplasia, 5 advanced adenomas, and 10 CRCs. Meta-analysis of these studies showed that diverticulosis was not associated with an advanced colorectal neoplasia (OR 0.98, 95% CI 0.63–1.50, *I*^2^ = 96%) (Fig 2).

### Diverticulosis and colorectal adenomas and polyps

**Fig 3:**
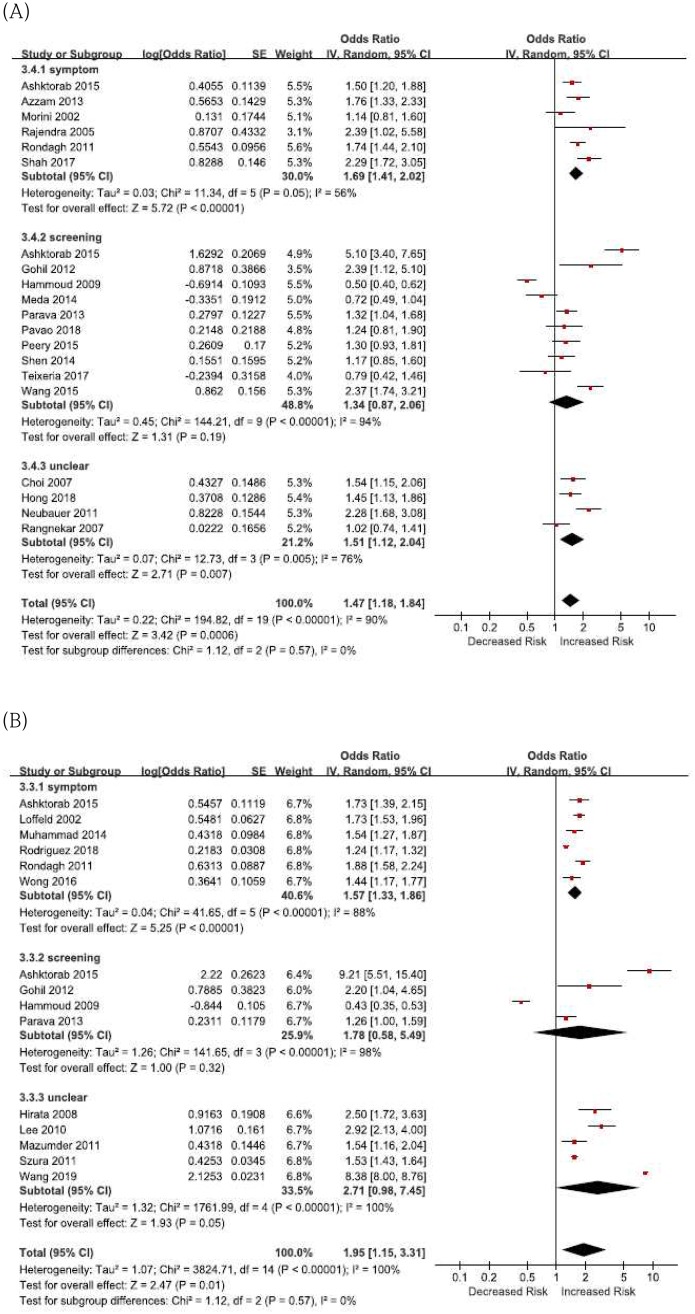
Forest pots of diverticulosis with colorectal adenomas and polyps. (A) diverticulosis with colorectal adenomas (B) diverticulosis with colorectal polyps.

Nineteen studies investigated the association between diverticulosis and adenomas, and meta-analysis found that diverticulosis was significantly related with an increased risk of adenomas (OR 1.47, 95% CI 1.18–1.84, *I*^2^ = 90%) (Fig 3A). Fourteen studies reported the association between diverticulosis and polyps. Meta-analysis showed a positive correlation between diverticulosis and polyps (OR 1.95, 95% CI 1.15–3.31, *I*^2^ = 100%) (Fig 3B). Multivariate analysis also revealed similar associations, showing diverticulosis with increased risks of adenomas (OR 1.76, 95% CI 1.34–2.32, *I*^2^ = 65%) and polyps (OR 1.90, 95% CI 1.50 ‒ 2.42, *I*^2^ = 57%) (S1 and S2 Figs).

We further performed subgroup analysis to evaluate whether the association differed based on the indication for colonoscopy. Of the 10 studies of patients who underwent screening colonoscopy, diverticulosis did not increase the risk of adenomas (OR 1.34, 95% CI 0.87–2.06, *I*^2^ = 94%) (Fig 3A). Similarly, 4 studies of patients with screening colonoscopy did not show increased risk for diverticulosis in relation to polyps (OR 1.78, 95% CI 0.58–5.49, *I*^2^ = 98%) (Fig 3B).

When focusing on serrated polyps, 3 studies reported the association between diverticulosis and serrated polyps [10, 13, 25]. Diverticulosis was associated with a higher risk of serrated polyps but did not increase the risk of serrated polyps in screening colonoscopy which were in line with the results in polyps (S3 Fig).

### Sensitivity analysis and evaluation of publication bias

**Fig 4:**
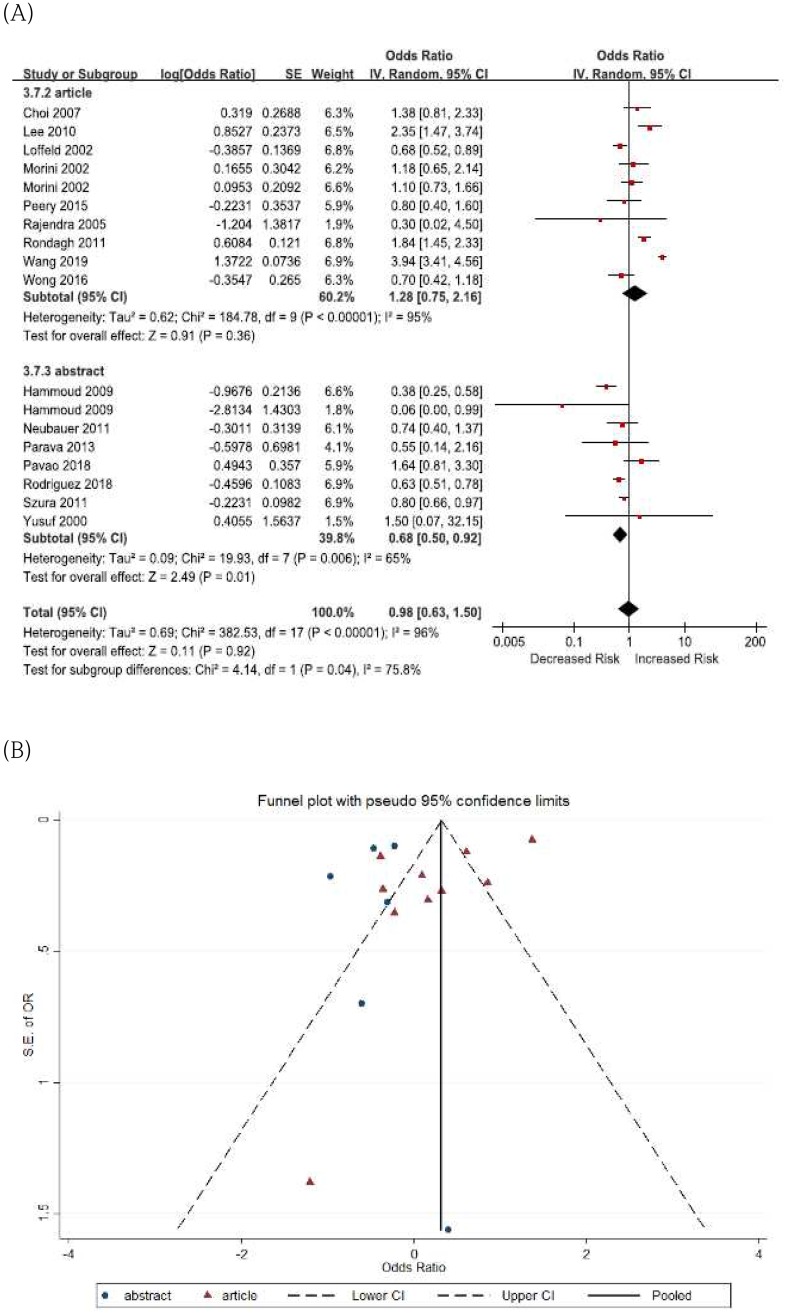
Sensitivity analysis and funnel plots for the association of diverticulosis with advanced neoplasia. (A) sensitivity analysis (B) funnel plots.

**Fig 5:**
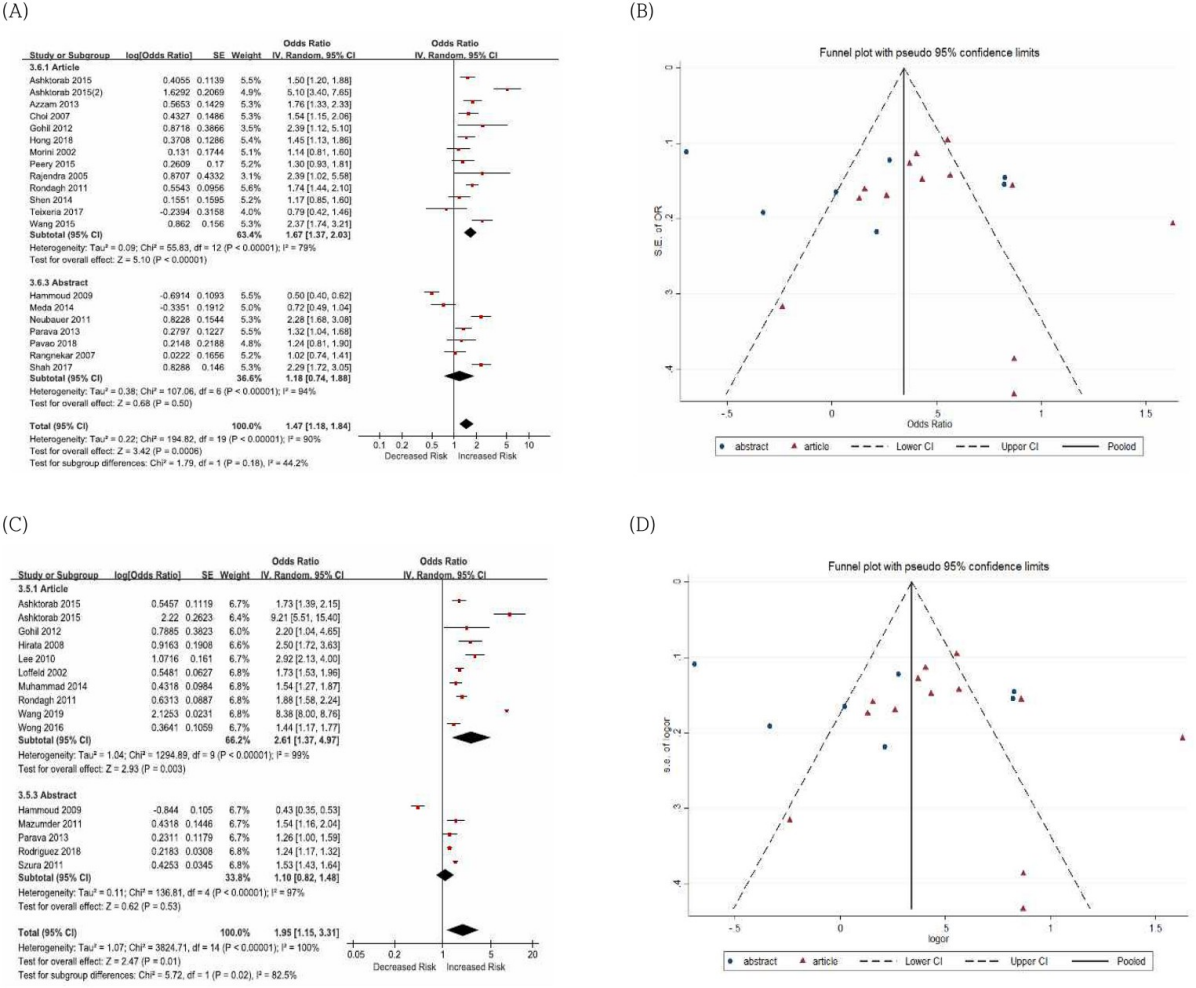
Sensitivity analysis and funnel plots for the association of diverticulosis with colorectal adenomas and polyps. (A, B) diverticulosis with colorectal adenomas (C, D) diverticulosis with colorectal polyps.

Finally, we conducted a sensitivity analysis by deleting abstract-only studies to measure the stability of our results. The pooled ORs of advanced colorectal neoplasia in patients with diverticulosis were significantly different according to publication types (articles, OR 1.28, 95% CI 0.75–2.16 vs. abstract-only studies, OR 0.68, 95% CI 0.50–0.92, *I*^2^ = 75.8%) (Fig 4A). Statistically similar results were obtained for adenomas in patients with diverticulosis (articles, OR 1.67, 95% CI 1.37–2.03 vs. abstract-only studies, OR 1.18, 95% CI 0.74–1.88, *I*^2^ = 44.2%) and polyps (articles, OR 2.61, 95% CI 1.37–4.97 vs. abstract-only studies, OR 1.10, 95% CI 0.82–1.48, *I*^2^ = 82.5%) (Fig 5A and 5C).

When we analyzed the association between diverticulosis and adenomas in screening colonoscopy according to the publication types, the pooled ORs of adenomas in patients with diverticulosis were also significantly different according to publication types (articles, OR 1.99, 95% CI 1.11–3.59 vs. abstract-only studies, OR 0.92, 95% CI 0.59–1.43, *I*^*2*^ = 76.4%) (S4 Fig).

According to the Egger test, there was no evidence of funnel plots asymmetry between the diverticulosis and advanced colorectal neoplasia, adenomas, and polyps (Figs 4B, 5B and 5D).

## Discussion

In this meta-analysis, we combined evidence from 29 cross-sectional studies involving 450,953 patients to investigate the association between diverticulosis and colorectal neoplasia. We found that diverticulosis was not associated with advanced colorectal neoplasia. Though diverticulosis was associated with a higher risk of adenomas and polyps, the risk of adenomas was not increased in patients with diverticulosis who underwent screening colonoscopy. Moreover, the increased risk of colorectal neoplasia in patients with diverticulosis was observed only in published studies and not in unpublished ones.

The pathologic mechanisms involved in both diverticulosis and colorectal neoplasia have been found to include diets low in dietary fiber and rich in saturated fat, and slow colonic transit times [6, 8, 41]. With increasing age, a deficiency in dietary fiber can lead to increases in abnormal movement by the colon as a result of abnormal thickening of the muscles in the colonic wall. The associated high intraluminal colonic pressure is believed to contribute to the development of diverticulosis, resulting in the prolongation of the time that the mucosa is in contact with potential carcinogens such as saturated fat and bile salts, and which is thought to contribute to colorectal neoplasia [3, 5]. In addition to the macroscopic changes in the colon, chronic low-grade microscopic inflammation and structural alterations in the extracellular matrix of patients with diverticulosis might lead to higher risk of the development of CRC [42, 43]. Therefore, many epidemiologic studies have been conducted to clarify the association between diverticulosis and colorectal neoplasia. However, despite the common potential mechanisms of pathogenesis for diverticulosis and colorectal neoplasia, whether or not diverticulosis increases the risk of colorectal neoplasia remains controversial.

In 2008, a systematic review was performed to investigate the possible association between diverticulosis and colorectal neoplasia [44]. Although the results of some studies suggested an increased risk of left-sided CRC for patients with diverticulosis [45, 46], the overall risk of CRC was not increased compared to controls. Most studies on adenomas reported a positive correlation with diverticulosis [16, 17, 32, 47]; however, they could not draw conclusions on the association, because of the heterogenous study designs and settings of the individual studies. One of the major concerns lied in the vague definitions of outcomes. A Swedish cohort study reported a 1.8-fold increased risk of left-sided CRC in patients with diverticular disease compared with general populations [45]. However, the prevalence might have been overestimated because they included patients with a history of either diverticulitis or diverticulosis, and the current practice guidelines recommended colonoscopy to exclude CRC after an episode of acute diverticulitis [48]. Another concern was the representativeness of the population in studies targeting patients with extensive diverticulosis, which tended to report a higher prevalence of adenoma in the patients with diverticulosis than in the controls [14, 47]. To overcome these drawbacks, we focused on the studies that only evaluated the association between diverticulosis and colorectal neoplasia.

Our meta-analysis demonstrated that diverticulosis was not associated with an increased risk of advanced neoplasia. It is in agreement with two nationwide population-based studies reporting that except for the first year after the diagnosis of diverticular disease, diverticular disease did not appear to be associated with an increased risk of subsequent CRC [49, 50]. They suggested that the increased risk of CRC within the first year (adjusted hazard ratio (HR) 4.54 and OR 31.49) might be attributed to the misclassification of patients with CRC as having diverticular disease, and the screening effect of the more rigorous examinations for the case group than those performed for the comparison group in a cohort study. However, a recent meta-analysis by Jaruvongvanich et al. offered conflicting results. They found a 1.36-fold increased risk of CRC in patients with diverticulosis, although the finding did not reach statistical significance [51]. The discrepant results might be explained by the different modalities used to document diverticulosis. Diverticulosis and colorectal neoplasia were diagnosed not only by colonoscopy but also by barium enema and computed tomography colonography. In addition the International Classification of Diseases codes were used, which could lead to an overestimation of the association. Based on the currently available data, though limited, the diverticulosis could not be considered to increase the risk of advanced neoplasia including CRC.

In contrast, our data revealed that patients with diverticulosis had a 1.47-fold risk of colorectal adenomas and a 1.95-fold increased risk of polyps compared to controls without diverticulosis. Indeed, many studies reported a positive correlation between diverticulosis and adenomas [10, 13, 17, 32, 44, 51] and some authors have suggested that diverticulosis might be a risk factor for premalignant colorectal lesions, and that endoscopists performing a colonoscopy for a patient with diverticulosis should be aware of possible risk [10, 13]. However, when we performed subgroup analysis based on the indication for colonoscopy, an increased risk of adenomas and polyps was only found in patients who underwent colonoscopy because of their gastrointestinal symptoms and this trend was also reproduced in multivariate analysis. These results indicate that the tendency of symptomatic patients to undergo intensive colonoscopy might affect the rate of detection of adenoma. Therefore, our data are not yet conclusive to support the policy of frequent or intensive screening and/or surveillance colonoscopy in patients with diverticulosis. Future study regarding the effect of diverticulosis on adenomas and advanced colorectal neoplasia focusing on the patients with screening colonoscopy is needed.

It is noteworthy that the associations between diverticulosis and colorectal neoplasia were clearly varied, based on the type of publication. Diverticulosis was associated with an increased risk of adenomas and polyps in published studies, but no association was found in unpublished data. Studies of patients who underwent colonoscopy due to gastrointestinal symptoms were all published, in contrast, regarding screening colonoscopy, the risk were obviously different according to publication types. A recent meta-analysis pointed out their drawback of publication bias because funnel plots are asymmetric that could interfere with the interpretation of results [51]. Our review included all the available unpublished abstract-only studies (41.4%) to exclude publication bias, and demonstrated that the effect size of published data might be overestimated to draw skewed conclusions.

Our study has several limitations. First, since all the included studies in our meta-analysis were cross-sectional studies, the causal relationship between diverticulosis and colorectal neoplasia cannot be assessed. Second, adenoma represented the principal precursor to CRC [52], but also serrated polyps have been recognized as a contributor to CRC via serrated pathway [53]. However, in our meta-analysis, most of polyps were defined morphologically regardless of histologic evaluation. Though 3 studies about serrated polyps reported the results which were consistent with those in polyps, further studies are needed to clarify the association between diverticulosis and serrated polyps. Third, although the quality of a colonoscopy is well known to be related with the detection rate of adenoma [54], except for 2 of the studies, most of the included studies did not report the quality of their colonoscopies. Detection bias might have been a limitation of this study. Fourth, limited by the data in the reported studies, we could not obtain sufficient data on such confounding factors as dietary habits, fiber intake, and physical activity, as well as a family history of CRC. However, we performed multivariate analysis of the confounding factors included in individual studies on adenomas and polyps. In addition, we included abstract-only studies in our review which had a weakness to provide limited information than published articles. However, by contacting original authors about unpublished data, the ratio of unclearly reported results was not different according to the publication type. Finally, our meta-analysis has limitations similar to other meta-analyses because of the heterogeneity of the published data. However, by performing sensitivity analysis, we revealed that the effect size could be reported biased according to the publication type.

In conclusion, our meta-analysis demonstrated that diverticulosis was not associated with an increased risk of advanced colorectal neoplasia. Although diverticulosis is associated with a higher risk of adenomas and polyps, the increased risk is only observed in patients with gastrointestinal symptoms and not in screening populations. In addition, the effect of diverticulosis on colorectal neoplasia in currently available studies might be overestimated to get a conclusion. A prospective cohort study to elucidate not only the association of diverticulosis with colorectal neoplasia but also the causality of the two diseases is warranted.

## Supporting information

S1 Fig Forest plots of diverticulosis with colorectal adenomas by multivariate analysis. (TIF)

S2 Fig Forest plots of diverticulosis with colorectal polyps by multivariate analysis. (TIF)

S3 Fig Forest plots of diverticulosis with serrated polyps according to the indication for colonoscopy. (TIF)

S4 Fig Forest plots of diverticulosis with colorectal adenoma in screening colonoscopy according to the publication types. (TIF)

S1 Table (DOCX)

S1 Appendix (DOCX)
