# Supplementary figures and images for: The relationship between diverticulosis and colorectal neoplasia: A meta-analysis
Source: PLoS One. 2019 May 29;14(5):e0216380. doi: 10.1371/journal.pone.0216380 (PMC6541260; doi:10.1371/journal.pone.0216380)

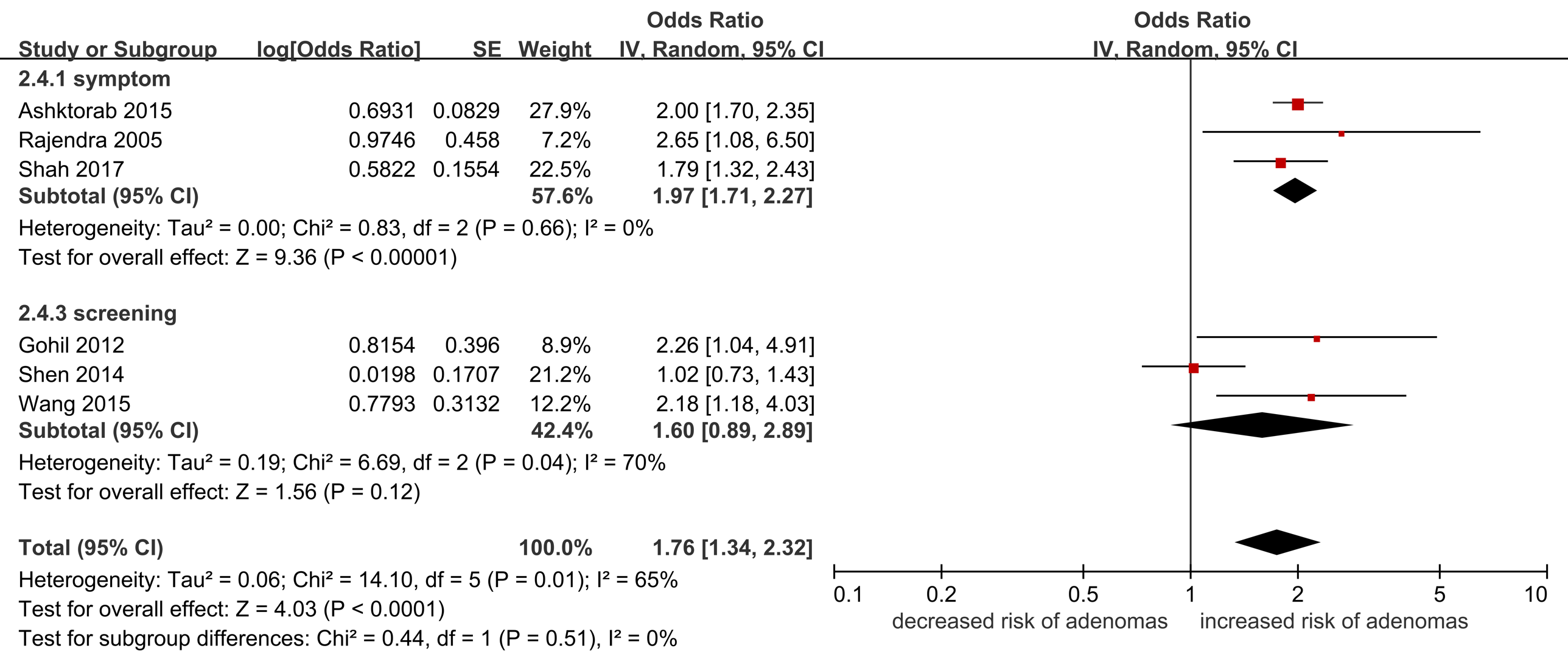

Supplement: S1 Fig — (TIF) [file pone.0216380.s001.tif]

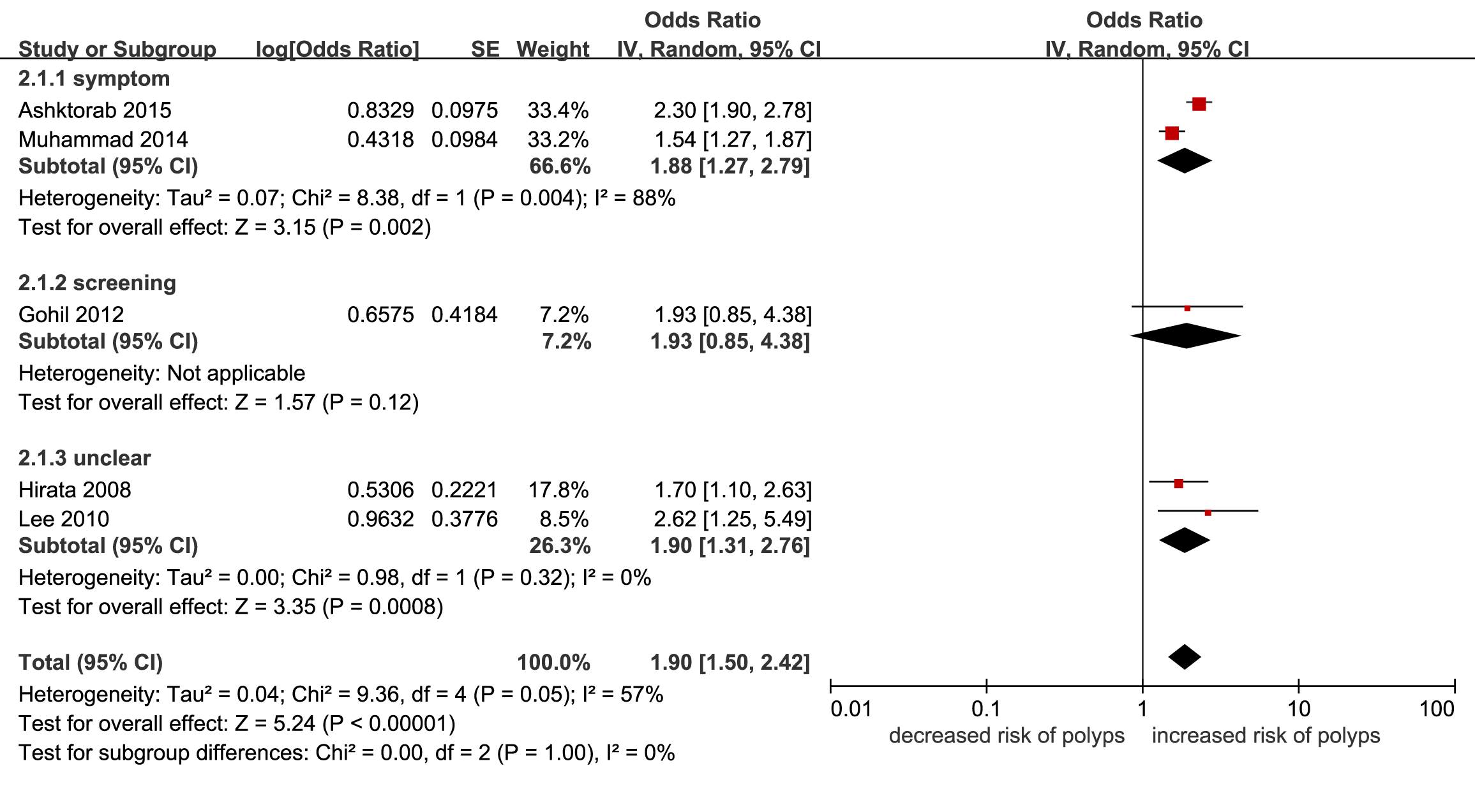

Supplement: S2 Fig — (TIF) [file pone.0216380.s002.tif]
