# Supplementary material for: The relationship between diverticulosis and colorectal neoplasia: A meta-analysis
Source: PLoS One. 2019 May 29;14(5):e0216380. doi: 10.1371/journal.pone.0216380 (PMC6541260; doi:10.1371/journal.pone.0216380)
Supplement: S1 Table — (DOCX) [file pone.0216380.s005.docx]

**Supporting information**

S1 Table. Quality assessment of included studies with the appraisal tool for cross-sectional studies (AXIS tool)

|  | Loffeld (2002) | Morini (2002) | Rajendra (2005) | Choi (2007) | Hirata (2008) | Lee (2010) | Randagh (2011) | Gohil (2012) | Azzam (2013) |
| --- | --- | --- | --- | --- | --- | --- | --- | --- | --- |
| Introduction |  |  |  |  |  |  |  |  |  |
| 1. Were the aims/objectives of the study clear? | Yes | Yes | Yes | Yes | Yes | Yes | Yes | Yes | Yes |
| Methods |  |  |  |  |  |  |  |  |  |
| 2. Was the study design appropriate for the stated aim(s)? | Yes | Yes | Yes | Yes | Yes | Yes | Yes | Yes | Yes |
| 3. Was the sample size justified? | Yes | Yes | Yes | Yes | Yes | Yes | Yes | Yes | Yes |
| 4. Was the target/reference population clearly defined? (Is it clear who the research was about?) | Yes | Yes | Yes | Yes | Yes | Yes | Yes | Yes | Yes |
| 5. Was the sample frame taken from an appropriate population base so that it closely represented the target/reference population under investigation? | Yes | Yes | Yes | Yes | Yes | Yes | Yes | Yes | Yes |
| 6. Was the selection process likely to select subjects/participants that were representative of the target/reference population under investigation? | No | Yes | No | Yes | Yes | Yes | Yes | No | Yes |
| 7. Were measures undertaken to address and categorise non-responders? | No | No | No | Yes | No | Yes | Yes | Yes | No |
| 8. Were the risk factor and outcome variables measured appropriate to the aims of the study? | Yes | Yes | Yes | Yes | Yes | Yes | Yes | Yes | Yes |
| 9. Were the risk factor and outcome variables measured correctly using instruments/measurements that had been trialled, piloted or published previously? | Yes | Yes | Yes | Yes | Yes | Yes | Yes | Yes | Yes |
| 10. Is it clear what was used to determined statistical significance and/or precision estimates? (eg, p values, CIs) | Yes | Yes | Yes | Yes | Yes | Yes | Yes | Yes | Yes |
| 11. Were the methods (including statistical methods) sufficiently described to enable them to be repeated? | Yes | Yes | Yes | Yes | Yes | Yes | Yes | Yes | Yes |
| Results |  |  |  |  |  |  |  |  |  |
| 12. Were the basic data adequately described? | Yes | Yes | Yes | Yes | Yes | Yes | Yes | Yes | Yes |
| 13. Does the response rate raise concerns about non-response bias? | No | No | No | Yes | No | Yes | Yes | Yes | No |
| 14. If appropriate, was information about non-responders described? | No | No | No | Yes | No | Yes | Yes | Yes | No |
| 15. Were the results internally consistent? | Yes | Yes | Yes | Yes | Yes | Yes | Yes | Yes | Yes |
| 16. Were the results for the analyses described in the methods, presented? | Yes | Yes | Yes | Yes | Yes | Yes | Yes | Yes | Yes |
| Discussion |  |  |  |  |  |  |  |  |  |
| 17. Were the authors’ discussions and conclusions justified by the results? | No | No | Yes | No | Yes | Yes | Yes | No | No |
| 18. Were the limitations of the study discussed? | Yes | No | Yes | Yes | No | Yes | Yes | Yes | Yes |
| Other |  |  |  |  |  |  |  |  |  |
| 19. Were there any funding sources or conflicts of interest that may affect the authors’ interpretation of the results? | No | No | Yes | No | No | Yes | Yes | Yes | Yes |
| 20. Was ethical approval or consent of participants attained? | Yes | No | Yes | No | No | Yes | Yes | Yes | Yes |

(continued)

|  | Muhammad (2014) | Ashktorab (2015) | Peery (2015) | Wang (2015) | Wong (2016) | Teixeria (2017) | Hong (2018) | Wang (2019) |
| --- | --- | --- | --- | --- | --- | --- | --- | --- |
| Introduction |  |  |  |  |  |  |  |  |
| 1. Were the aims/objectives of the study clear? | Yes | Yes | Yes | Yes | Yes | Yes | Yes | Yes |
| Methods |  |  |  |  |  |  |  |  |
| 2. Was the study design appropriate for the stated aim(s)? | Yes | Yes | Yes | Yes | Yes | Yes | Yes | Yes |
| 3. Was the sample size justified? | Yes | Yes | Yes | Yes | Yes | Yes | Yes | Yes |
| 4. Was the target/reference population clearly defined? (Is it clear who the research was about?) | Yes | Yes | Yes | Yes | Yes | Yes | Yes | Yes |
| 5. Was the sample frame taken from an appropriate population base so that it closely represented the target/reference population under investigation? | Yes | Yes | Yes | Yes | Yes | Yes | Yes | Yes |
| 6. Was the selection process likely to select subjects/participants that were representative of the target/reference population under investigation? | Yes | No | Yes | Yes | Yes | No | Yes | No |
| 7. Were measures undertaken to address and categorise non-responders? | No | No | No | No | Yes | No | Yes | No |
| 8. Were the risk factor and outcome variables measured appropriate to the aims of the study? | Yes | Yes | Yes | Yes | Yes | Yes | Yes | Yes |
| 9. Were the risk factor and outcome variables measured correctly using instruments/measurements that had been trialled, piloted or published previously? | Yes | Yes | Yes | Yes | Yes | Yes | Yes | Yes |
| 10. Is it clear what was used to determined statistical significance and/or precision estimates? (eg, p values, CIs) | Yes | Yes | Yes | Yes | Yes | Yes | Yes | Yes |
| 11. Were the methods (including statistical methods) sufficiently described to enable them to be repeated? | Yes | Yes | Yes | Yes | Yes | Yes | Yes | Yes |
| Results |  |  |  |  |  |  |  |  |
| 12. Were the basic data adequately described? | Yes | Yes | Yes | Yes | Yes | Yes | Yes | Yes |
| 13. Does the response rate raise concerns about non-response bias? | No | No | No | No | Yes | No | Yes | No |
| 14. If appropriate, was information about non-responders described? | No | No | No | No | Yes | No | Yes | No |
| 15. Were the results internally consistent? | Yes | Yes | Yes | Yes | Yes | Yes | Yes | Yes |
| 16. Were the results for the analyses described in the methods, presented? | No | Yes | Yes | Yes | Yes | Yes | No | Yes |
| Discussion |  |  |  |  |  |  |  |  |
| 17. Were the authors’ discussions and conclusions justified by the results? | No | Yes | Yes | Yes | No | No | Yes | No |
| 18. Were the limitations of the study discussed? | Yes | Yes | Yes | Yes | No | Yes | Yes | No |
| Other |  |  |  |  |  |  |  |  |
| 19. Were there any funding sources or conflicts of interest that may affect the authors’ interpretation of the results? | Don't know | Yes | Yes | Yes | No | No | Yes | No |
| 20. Was ethical approval or consent of participants attained? | Yes | Yes | Yes | Yes | Yes | Yes | Yes | Yes |

*Quality assessment were not available in abstract only studies
