# Supplementary material for: The relationship between diverticulosis and colorectal neoplasia: A meta-analysis
Source: PLoS One. 2019 May 29;14(5):e0216380. doi: 10.1371/journal.pone.0216380 (PMC6541260; doi:10.1371/journal.pone.0216380)
Supplement: S1 Appendix — (DOCX) [file pone.0216380.s006.docx]

**Supporting information**

*S1 Appendix*

MEDLINE

1. Diverticulosis[tiab] OR Diverticular[tiab] OR Diverticulum[tiab] OR Diverticulitis[tiab] OR diverticula[tiab] 26555
2. (("Diverticulosis, Colonic"[Mesh]) OR "Diverticulum"[Mesh:NoExp]) OR "Diverticulum, Colon"[Mesh] 14969
3. 1 OR 2 28955
4. (Colorectal[tiab] OR Colonic[tiab] OR colon[tiab] OR Cecum[tiab] OR Cecal[tiab] OR Rectal[tiab] OR rectum[tiab]) AND (malignant[tiab] OR Neoplasia[tiab] OR Neoplasm[tiab] OR cancer[tiab] OR carcinoma[tiab] OR adenocarcinoma[tiab] OR cancers[tiab] OR Neoplasms[tiab] OR carcinomas[tiab] OR adenocarcinomas[tiab] OR tumor[tiab] OR tumors[tiab] OR tumour[tiab] OR tumours[tiab] OR malignancy[tiab] OR Polyps[tiab] OR Polyp[tiab] OR Adenoma[tiab] OR Neoplastic[tiab]) 241896
5. (((("Colorectal Neoplasms"[Mesh]) OR "Cecal Neoplasms"[Mesh]) OR "Colonic Polyps"[Mesh]) OR "Adenoma, Villous"[Mesh]) OR "Adenomatous Polyps"[Mesh] 194315
6. 4 OR 5 286262
7. 3 AND 6 3100
8. 7 NOT "review"[Publication Type] OR "review literature as topic"[MeSH Terms] 2740

EMBASE

1. Diverticulosis:ab,ti OR Diverticular:ab,ti OR Diverticulum:ab,ti OR Diverticulitis:ab,ti OR diverticula:ab,ti 34201
2. 'colon diverticulosis'/exp OR 'diverticulosis'/de OR 'diverticulitis'/exp 25848
3. 1 OR 2 40907
4. (Colorectal:ab,ti OR Colonic:ab,ti OR colon:ab,ti OR Cecum:ab,ti OR Cecal:ab,ti OR Rectal:ab,ti OR rectum:ab,ti) AND (malignant:ab,ti OR Neoplasia:ab,ti OR Neoplasm:ab,ti OR cancer:ab,ti OR carcinoma:ab,ti OR adenocarcinoma:ab,ti OR cancers:ab,ti OR Neoplasms:ab,ti OR carcinomas:ab,ti OR adenocarcinomas:ab,ti OR tumor:ab,ti OR tumors:ab,ti OR tumour:ab,ti OR tumours:ab,ti OR malignancy:ab,ti OR Polyps:ab,ti OR Polyp:ab,ti OR Adenoma:ab,ti OR Neoplastic:ab,ti) 348548
5. 'colon tumor'/exp OR 'rectum tumor'/exp OR 'cecum tumor'/exp OR 'adenomatous polyp'/exp 345414
6. 4 OR 5 434016
7. 3 AND 6 6036
8. 7 NOT ('human cell'/de OR 'human tissue'/de OR 'nonhuman'/de) 5326
9. 8 NOT ('conference review'/it OR 'review'/it) 4813

COCHRANE

1. Diverticulosis or Diverticular or Diverticulum or Diverticulitis or diverticula:ti,ab,kw 762
2. MeSH descriptor: [Diverticulosis, Colonic] explode all trees 87
3. MeSH descriptor: [Diverticulum] this term only 38
4. MeSH descriptor: [Diverticulum, Colon] explode all trees 37
5. 2-4/OR 148
6. 1 OR 5 762
7. (Colorectal or Colonic or colon or Cecum or Cecal or Rectal or rectum) and (malignant or Neoplasia or Neoplasm or cancer or carcinoma or adenocarcinoma or cancers or Neoplasms or carcinomas or adenocarcinomas or tumor or tumors or tumour or tumours or malignancy or Polyps or Polyp or Adenoma or Neoplastic):ti,ab,kw 18257
8. MeSH descriptor: [Colorectal Neoplasms] explode all trees 6916
9. MeSH descriptor: [Cecal Neoplasms] explode all trees 16
10. MeSH descriptor: [Colonic Polyps] explode all trees 389
11. MeSH descriptor: [Adenoma, Villous] explode all trees 6
12. MeSH descriptor: [Adenomatous Polyps] explode all trees 199
13. 8-12/OR 7087
14. 7 OR 13 18364
15. 6 AND 14 224
16. 15/TRIALS 208

Web of Science

1. TOPIC: (Diverticulosis OR Diverticular OR Diverticulum OR Diverticulitis OR diverticula) OR TITLE: (Diverticulosis OR Diverticular OR Diverticulum OR Diverticulitis OR diverticula) 21884
2. TOPIC: ((Colorectal OR Colonic OR colon OR Cecum OR Cecal OR Rectal OR rectum) AND (malignant OR Neoplasia OR Neoplasm OR cancer OR carcinoma OR adenocarcinoma OR cancers OR Neoplasms OR carcinomas OR adenocarcinomas OR tumor OR tumors OR tumour OR tumours OR malignancy OR Polyps OR Polyp OR Adenoma OR Neoplastic)) OR TITLE: ((Colorectal OR Colonic OR colon OR Cecum OR Cecal OR Rectal OR rectum) AND (malignant OR Neoplasia OR Neoplasm OR cancer OR carcinoma OR adenocarcinoma OR cancers OR Neoplasms OR carcinomas OR adenocarcinomas OR tumor OR tumors OR tumour OR tumours OR malignancy OR Polyps OR Polyp OR Adenoma OR Neoplastic)) 332696
3. 1 AND 2 2031
4. 3 Refined by: [excluding] DOCUMENT TYPES: ( REVIEW ) 1868

SCOPUS

1. TITLE-ABS-KEY ( diverticulosis OR diverticular OR diverticulum OR diverticulitis OR diverticula ) 41673
2. ( INDEXTERMS ( "Diverticulosis, Colonic" ) ) OR ( INDEXTERMS ( "Diverticulum" ) ) OR ( INDEXTERMS ( "Diverticulum, Colon" ) ) 19788
3. 1 OR 2 41673
4. TITLE-ABS-KEY ( ( colorectal OR colonic OR colon OR cecum OR cecal OR rectal OR rectum ) AND ( malignant OR neoplasia OR neoplasm OR cancer OR carcinoma OR adenocarcinoma OR cancers OR neoplasms OR carcinomas OR adenocarcinomas OR tumor OR tumors OR tumour OR tumours OR malignancy OR polyps OR polyp OR adenoma OR neoplastic ) ) 391478
5. ( INDEXTERMS ( "Colorectal Neoplasms" ) ) OR ( INDEXTERMS ( "Cecal Neoplasms" ) ) OR ( INDEXTERMS ( "Colonic Polyps" ) ) OR ( INDEXTERMS ( "Adenoma, Villous" ) ) OR ( INDEXTERMS ( "Adenomatous Polyps" ) ) 85015
6. 4 OR 5 393203
7. 3 AND 6 6015
8. 7 EXCLUDE ( DOCTYPE , "re" ) 5309
